# Efficient and scalable scaffolding using optical restriction maps

**DOI:** 10.1186/1471-2164-15-S5-S5

**Published:** 2014-07-14

**Authors:** Subrata Saha, Sanguthevar Rajasekaran

**Affiliations:** 1 Department of CSE, UCONN, Storrs, USA; 2 Department of CSE, UCONN, Storrs, USA

**Keywords:** Next Generation Sequencing (NGS), Optical Restriction Map (ORM), Human Genome Project (HGP), String Graph Assembler (SGA), Contigs, Scaffolding

## Abstract

In the next generation sequencing techniques millions of short reads are produced from a genomic sequence at a single run. The chances of low read coverage to some regions of the sequence are very high. The reads are short and very large in number. Due to erroneous base calling, there could be errors in the reads. As a consequence, sequence assemblers often fail to sequence an entire DNA molecule and instead output a set of overlapping segments that together represent a consensus region of the DNA. This set of overlapping segments are collectively called contigs in the literature. The final step of the sequencing process, called scaffolding, is to assemble the contigs into a correct order. Scaffolding techniques typically exploit additional information such as mate-pairs, pair-ends, or optical restriction maps. In this paper we introduce a series of novel algorithms for scaffolding that exploit optical restriction maps (ORMs). Simulation results show that our algorithms are indeed reliable, scalable, and efficient compared to the best known algorithms in the literature.

## Introduction

To conduct basic biological research such as but not limited to diagnostic, biotechnology, forensic biology, biological pathways and knowledge of DNA sequences has become inevitable. Scientists need to know the sequence of bases to reveal genetic information that is hidden in a particular segment of a DNA molecule. For example, they can use sequence information to identify which stretches of DNA molecule contain genes, as well as analyze those genes to detect potential changes in the sequence that may cause diseases. So, to obtain an in-depth knowledge of a particular DNA molecule, sequencing of that molecule is the primary step. DNA sequencing is any process that is used to map out the precise order of the nucleotides within a single strand of a DNA molecule. The structure of DNA was modeled as a double helix in 1953. The first notable method for sequencing DNA was developed during the 1970s known as Sanger sequencing. It is a method of DNA sequencing based on the selective incorporation of chain-terminating dideoxynucleotides by DNA polymerase during in vitro DNA replication [6,7]. It was developed by Frederic Sanger and his colleagues in 1977 and was the most widely used sequencing technology until the advent of NGS technologies. An alternative to Sanger was shotgun sequencing [8,9]. By the time the Human Genome Project (HGP) began in 1990, only a few scientific laboratories had the ability to sequence a mere 100k bases, and the total cost of sequencing remained very high. Since then, technological improvements and computerized automation have increased the sequencing speed and lowered the cost to the point where individual genes can be sequenced routinely, and some laboratories managed to sequence well over 100 million bases per year. Beginning in the late 1990s, the scientific community has developed a number of new DNA sequencing technologies including the first of the "next-generation" sequencing methods.

High-throughput or next-generation sequencing technologies parallelizes the sequencing process and produce thousands or millions of short reads (25-100 bp) simultaneously at a single run. Some of the sequencing technologies dominating the NGS market today are Massively parallel signature sequencing (MPSS), 454 pyrosequencing, Illumina (Solexa) sequencing, SOLiD sequencing, Ion semiconductor sequencing, etc. An introductory review of these techniques can be found in [1]. After generating NGS reads, they can either be assembled *de novo *or aligned to a known reference sequence [2]. The choice solely depends on the biological application of interest as well as cost, effort, and time constraints. For example, if the intended application of interest is to determine a complete genomic sequence of a new species, we have to follow *de novo *sequencing strategy. On the contrary, identifying genetic variations in multiple strains of highly related genomes can be accomplished by aligning NGS reads to their reference genomes. This approach is cheaper and faster than *de novo *sequencing. But there are some limitations and challenges associated with this alignment approach. One of the most important challenges is in placing the reads within repetitive regions in the reference genome. Besides this, some of the regions existing in the source genome may not even exist in the reference genome. This could happen because of gaps in the reference genome [3]. The problem of aligning reads in repetitive regions can be solved by exploiting mate-pair reads information. *De novo *sequencing techniques also face challenges in repetitive regions and from low read coverages that result in gaps in the constructed sequence. The former can be overcome by employing mate-pair reads [4] or optical restrictions maps [5] information and the later can be solved increasing the read coverage.

In sequencing DNA is first shredded randomly into numerous smaller fragments. The resulting fragments are sequenced using the chain termination method to obtain reads. Multiple overlapping reads for the target DNA are obtained by performing several rounds of this fragmentation and sequencing. The resulting reads of these fragments are then reassembled into their original order based on overlaps. Reassembly is done by a computer program ultimately yielding the complete and continuous sequence. A contig is a series of overlapping DNA sequences used to make a physical map that reconstructs the original DNA sequence of a chromosome or a region of a chromosome. It is a set of overlapping DNA segments that together represent a consensus region of DNA. If the coverage is large enough and the sequenced reads are error free, there should be only one contig containing the entire genome. But in the next generation sequencing technologies the coverage can be low resulting in gaps and the reads also can be erroneous. As a consequence sequence assemblers typically produce multiple contigs. Obtaining the exact orientation and precise order of the contigs is the next challenging task. This step is known as scaffolding.

In genomic mapping, a scaffold is a series of contigs that are in the correct order but not necessarily connected in one continuous stretch of the genomic sequence. So, a scaffold is not only composed of contigs but also gaps. The problem of finding the correct order of the contigs can be posed as the problem of finding a permutation of these contigs that optimizes an objective criterion. Scaffolding is known to be NP-hard. Any information about the orderings such as the sizes of fragments of the DNA molecule can indeed help in devising an efficient algorithm for scaffolding. We can get fragment size information by employing restriction enzymes. A restriction enzyme (or restriction endonuclease) is an enzyme that cuts DNA at or near some specific recognition nucleotide sequences known as restriction sites. Restriction enzymes are of three types and found in bacteria and archaea. A restriction enzyme acts against invading viruses by electively cutting up a foreign DNA in a process called restriction. In general, restriction enzymes recognize a specific sequence of nucleotides and produce a double-stranded cut in the DNA. The recognition sequences usually vary between 4 and 8 nucleotides, and they are generally palindromic sequences. The locations of these specific sequences of nucleotides on a DNA molecule are called restriction sites. A restriction map detects known restriction sites within a sequence of DNA by cleaving it with a specific restriction enzyme. A restriction map provides a number of fragment sizes which collectively serve as a unique "fingerprint" or "barcode" for that sequence [10]. An optical restriction map (ORM) [11] is also similar to a restriction map with only one difference. It provides an ordered list of fragment sizes and this method has been combined with the assembly process to sequence whole genomes. Some of the recent research on ORMs in the context of contigs assembly can be found in [12], [13], [14], or [5].

We have employed ORMs in the context of scaffolding to find the relative order and correct placement of contigs produced by sequence assemblers. In this paper we propose several algorithms for scaffolding. We use a two phase strategy for scaffolding (just like the authors of [5]). In the first phase we compute a score for each contig corresponding to each possible placement of the contig in the ORM. In the second phase we utilize the scores computed in the first phase to come up with a non-overlapping placement of (possibly a subset of) the contigs in the ORM. In brief, we transform each contig into an ordered sequence of fragment sizes (just like the ORM). A greedy scoring scheme is then applied to find a score for each contig for each possible placement of the contig in the ORM. Greedy placement algorithms are then used to place the contigs in a correct order by using the matching scores. To validate the robustness of our proposed algorithms we have introduced different types of errors. Our simulation results on both real and synthetic data show that our algorithms are indeed scalable and efficient in terms of both accuracy and time. The rest of this paper is organized as follows: Section 2 contains the algorithms we propose. Simulation results and relevant discussions are presented in Section 3. Section 4 concludes the paper.

## Methods

There are two phases in our algorithm. In the first phase we compute a score for each contig corresponding to each possible placement of the contig in the ORM. In the second phase we utilize the scores computed in the first phase to come up with a non-overlapping placement of the contigs in the ORM. These two phases are described in Sections and, respectively.

### A scoring scheme

#### Overview

To effectively and correctly order the contigs we need a reliable scoring mechanism. As the genomic sequence can be composed of millions or even billions of characters, we should also consider the time spent by the proposed algorithms. There is a trade off between the time an algorithm takes and the accuracy it gives. We achieve a very good balance between these two. This is done by carefully formulating the scoring algorithm. For each contig, we generate an ordered list of fragment sizes. Since we know the sequence of the restriction enzyme from the ORM of the genomic sequence, we can easily find the ordered restriction fragment sizes of any contig by incorporating *in silico *digest of the restriction enzyme. The resulting list of ordered fragment sizes can be mapped with the ORM. Assuming that there are no errors either in the ORM or the fragment sizes of the contigs, for any given ordered fragment sizes of a contig, in general, there will exist a subset of matching ordered fragment sizes in the ORM. Exploiting this information we can order the contigs. But in a real world scenario the data often may not be error free. Errors could occur due to the omission of some restriction sites or a change in some fragment sizes (due to sequencing errors). To quantify the effect of the errors a scoring mechanism is introduced.

Let $A=\left\{c_{1}^{i},c_{2}^{i},\ldots ,c_{n_{i}}^{i}\right\}$ be the set of the ordered *in silico *fragment sizes of a contig *C_i _*and *B *= {*o_l_, o*_*l*+1_, *o*_*l*+2_, . . . , *o*_*m*−*l*+1_} be the set of ordered fragment sizes of a particular region of the ORM stretching from the *lth *fragment to the (*m − l *+ 1)*^th ^*fragment. The score of the contig *Ci *for the region stretching from the *lth *fragment to the (*m − l *+ 1)*^th ^*fragment of the ORM is defined as follows:

(1) $$Score\left(C_{i}\right)=\left|\overset{n_{i}}{\underset{j=1}{\sum }}c_{j}^{i}-\overset{m-l+1}{\underset{j=1}{\sum }}o_{j}\right|+P*MRS$$

where *P *and *M RS *are the penalty term and number of missed restriction sites, respectively. Penalty term *P *is user defined and should be very large. Under ideal circumstances where there are no errors in reads, there are no errors in the ORM, the assembly is perfect, etc., we should not tolerate any missed restriction sites. In this case *P *could be even *∞*. But in practice, depending on the technology employed, we could expect to see some errors in every process. As a result, we have to use a finite penalty. The value of *P *will thus depend on the error rates in the different technologies. If the expected error rate is low, then *P *has to be large. If the expected error rate is high, then *P *has to be low. In our experiments a value of 999 for *P *seems to yield good results.

More details on our algorithms are given in the next section.

#### A greedy scoring algorithm

The input to the *Greedy Scoring *algorithm are an ORM of the genomic sequence of interest, an ordered list of fragment sizes for each contig, and a penalty term. The fragment sizes may not be known exactly. Each fragment size in general can be thought of as a random variable for which we know the mean and the standard deviation. For simplicity assume that the standard deviation is the same (say *σ*) for all the fragment sizes. The algorithm proceeds greedily to calculate the score of each contig. In fact, the algorithm computes multiple scores for each contig. If *m *is the number of fragment sizes in the ORM, then the algorithm computes *m *scores for each contig.

Let *o*_1_, *o*_2_, . . . , *o_m _*be the fragment sizes in the ORM. Let *C *be any contig and let the fragment sizes of *C *be *c*_1_, *c*_2_, . . . , *c_n_*. A score for *C *is computed by matching *c*_1 _with *o*_1_; Another score is computed by matching *c*_1 _with *o*_2_; and so on. In other words, we compute a score for *C *by matching *c*_1 _with *o_i _*for each possible value of *i*, 1 *≤ i ≤ m*. What is the score when *c*_1 _is matched with *o_i _*(for some specific value of *i*)? We correlate a prefix of *C *(of minimum length) with a prefix of *o_i_, o*_*i*+1_, . . . , *o_m _*such that the two prefix sums are nearly the same (within *σ*). In other words, we identify the least integer *u *and an integer *q *such that $|\sum_{j=1}^{u}c_{i}-\sum_{j=1}^{i+q-1}o_{i}|\;\leq \sigma$. Once we find such *u *and *q*, we match *c_u _*with *o*_*i*+*q−*1_. Now we proceed recursively, i.e., we look for a prefix (of least length) of *c*_*u*+1_, *c*_*u*+1_, . . . , *c_n _*and a prefix of *o*_*i*+*q*_, *o*_*i*+*q*+1_, . . . , *o_m _*whose sums are nearly the same (up to *σ*); and so on.

The score for the resultant mapping of the contig *C *is obtained using Equation 1. For example, the partial score corresponding to the mapping of *c_u _*with *o*_*i+q*−1_ is $|\sum_{j=1}^{u}c_{i}-\sum_{j=1}^{i+q-1}o_{i}|+\left[\left(u-1\right)+\left(q-1\right)\right]*P$. Such partial scores are computed and added. Note that when we map *c*_1 _with *o_i_*, the last fragment *c_n _*of the contig will be mapped with some fragment *o_t _*in the ORM. Corresponding to this mapping of the contig *C*, we refer to *o_i _*as the starting fragment and *o_t _*as the ending fragment. For the base case when *i *= *m*, we match *c_n _*with *o_m_*. Also when $\sum_{j=1}^{n}c_{j}>\sum_{j=i}^{m}o_{j}$ we match *c_n _*with *o_m_*.

More details of the algorithm can be found in Algorithm 1. The run time of our greedy scoring algorithm is *O*(*mnr*), where *m *is the number of fragments in the optical map, *r *is the number of contigs and *n *is the maximum number of fragments in any contig.

### Placement schemes

The placement scheme utilizes the matching scores of the contigs to find the correct order. We propose three different placement algorithms that are described below.

#### Some notations

The list of ordered fragment sizes in the ORM is *o*_1_, *o*_2_, . . . , *o_m_*. The number of contigs is denoted as *r*. Let the cotigs be *C*_1_, *C*_2_, . . . , *C_r_*. The number of fragments in *C_i _*is denoted as *n_i_*, for 1 *≤ i ≤ r*. The list of ordered fragment sizes corresponding to *C_i _*is $c_{1}^{i},c_{2}^{i},\ldots ,c_{n_{i}}^{i}$, for 1 *≤ i ≤ r*. Let *k *denote ${\text{max}}_{i=1}^{r}n_{i}$.

#### Greedy placement algorithm 1 - GPA1

*GPA1 *takes as input the contigs and the ORM together with the output of Algorithm 1. If *m *is the number of ordered fragments in the ORM, then the number of scores associated with each contig will be *m*, as described in the previous section. The algorithm proceeds as follows: At first the matching scores associated with each contig are sorted individually in increasing order. The first position of the sorted list of each contig contains the minimum score among all the scores. As the penalty term is very large, this matching score is the best score for placing this contig anywhere in the ORM.

We now sort the contigs based on the indices of the starting fragments corresponding to the best scores. As an example, assume that there are 5 contigs $C_{1}^{\prime },C_{2}^{\prime },\ldots ,C_{5}^{\prime }$ and consider their best scores. For each such score there is a starting fragment and an ending fragment. If the starting fragments of these contigs are *o*_5_, *o*_11_, *o*_3_, *o*_22_, and *o*_7_, respectively, then the sorted order of the fragments will be *o*_3_, *o*_5_, *o*_7_, *o*_11_, and *o*_22_. So the corresponding contigs with respect to its starting fragments will be $C_{3}^{\prime },C_{1}^{\prime },C_{5}^{\prime },C_{2}^{\prime },C_{4}^{\prime }$. In general, let this sorted order be *C*_1_, *C*_2_, . . . , *C_r _*. Followed by this, we attempt to place the contigs in the ORM in this order (using the mapping corresponding to the best score). Specifically, we first try to place *C*_1_; Next we attempt to place *C*_2_; and so on. When we try to place any contig *C*, we check whether the starting and/or ending fragments of *C *will overlap with any of the already placed contigs. If there is such an overlap, we discard *C *and move onto the next contig in the sorted list.

A detailed pseudocode is supplied in Algorithm 2. Let *m *be the number of fragments in the optical map, and *r *be the number of contigs. Intuitively the number of matching scores of each contig *C_i _*is at most *O*(*m*). Since the matching score is an integer, sorting matching scores of each contig *C_i _*takes at most *O*(*m*) time. So, the execution time of lines 2-7 in Algorithm 2 is *O*(*mr*). Sorting contigs with respect to starting fragment takes *O*(*r*) time (line 8). In the worst case lines 9-12 take *O*(*r*^2^) time. Since *r *≪ *m*, the run time of Algorithm 2 is *O*(*mr*).

#### Greedy placement algorithm 2 - GPA2

*GPA2 *proceeds as follows: At first the matching scores associated with each contig are sorted individually in increasing order. Note that we consider *m *possible matchings for each contig and hence each contig has a list of *m *mappings and scores. Let the list of mappings (in sorted order of the matching scores) for contig *C *be *L_C_*.

The number of matching sites for a contig mapping is defined to be the number of fragments in the contig that are matched with fragments in the ORM. For each contig, we know that there are *m *scores (with one score per starting fragment or mapping). Corresponding to each starting fragment (i.e., mapping) we can also compute the number of matching sites. Thus for every contig, we have a list of *m *numbers of matching sites. We identify for each contig the mapping that has the largest number of matching sites. Let *b_C _*be this number for contig *C*. We order the contigs based their *b_C _*values in non-increasing order. Let the sorted list be $C_{1}^{\prime },C_{2}^{\prime },\ldots ,C_{r}^{\prime }$ based on their *b_C _*values.

Place the contigs one-by-one based on the above sorted list starting from $C_{1}^{\prime }$ For any contig *C*, mappings for this contig will be considered as per the list *L_C_*. In other words, the first time when we try to place *C*, we will use the mapping found in *L_C _*[1]. When we try to place *C *using this specific mapping, we check whether the starting and/or ending fragments of the contig will overlap with already placed contigs. If there is no overlap, we process the next contig. If there is an overlap while placing *C *(using the mapping in *L_C _*[1]), we move to the next entry in *L_C_*, i.e., *L_C _*[2]. If successful, we process the next contig. If not, we move on to the next entry in *L_C_*, and so on. We make repeated attempts to place *C *at most *d *times (where *d *is a user-specified parameter). If we are not successful in these *d *attempts, we ignore *C *and proceed to process the next contig.

Additional details of the algorithm are supplied in Algorithm 3. Let *m *be the number of fragments in the optical map, and *r *be the number of contigs. The run time of lines 2-7 in Algorithm 3 is *O*(*mr*) as discussed above. Sorting contigs with respect to the matched sites takes *O*(*r*) time (line 8). Lines 13-20 take *O*(*rd*) time. In line 21 sorting contigs with respect to starting fragment takes *O*(*r*) time. Since *d *≪ *r *≪ *m*, the run time of Algorithm 3 is *O*(*mr*).

**Algorithm 1: **Greedy Scoring

 **Input**: *OpticalRestrictionM ap*[1*..m*], *ContigF ragmentList*[1*..r*][1*..k*], *P enalty, P*

 **Output**: Contigs with associated scores *CS*[1*..r*][1*..m*]

 **begin**

**1 ***i ← *1

 **repeat**

**2 **  *j ← *0

**3 **  *case ← *0

   **repeat**

    set *matched_sites, contig_frag_size, op_frag_size *to 0

**4 **   *text_pos ← j *+ 1

**5 **   *pattern_pos, missed_res_sites *to 1

    **repeat**

**6     if **(case == 0){

**7 **      *contig_frag_size *= *ContigF ragmentList*[*i*][*pattern_pos*]

**8 **      *op_frag_size *= *OpticalRestrictionM ap*[*text pos*]

**9 **     } **else if **(case == 1){

**10 **      *contig_frag_size *+= *ContigF ragmentList*[*i*][*pattern_pos*]

**11 **     } **else if **(case == 2){

**12 **      *op_frag_size *+= *OpticalRestrictionM ap*[*text_pos*]

**13 **     }

**14 **     *lower_bound *= *op_frag_size − std*(*text_pos*)

**15 **     *upper_bound *= *op_frag_size *+ *std*(*text_pos*)

**16      if **(*con_frag_size ≥ lower_bound ***and**

      *con_frag_size ≤ upper_bound*){

**17 **      Increment *pattern_pos, text_pos*, and *matched_sites *by 1

**18 **      case = 0

**19 **     } **else if **(*con_frag_size < lower_bound*){

**20 **      Increment *pattern_pos*, and *missed_res_sites *by 1

**21 **      case = 1

**22 **     } **else if **(*con_frag_size > upper_bound*){

**23 **      Increment *text_pos*, and *missed_res_sites *by 1

**24 **      case = 2

**25 **     }

**26      if **(*pattern_pos ≥ |ContigF ragmentList*[*i*][1*..k*]*|*){

**27 **      Calculate score using Equation 1

**28 **      Insert the score along with the starting and ending positions in *CS*

**29 **     }

     **until ***pattern_pos ≤ |ContigFragmentList*[*i*][1*..k*]*|*;

**30 **    *j ← j *+ 1

    **until ***j ≤ m*;

**31 **   *i ← i *+ 1

**   until ***i ≤ r*;

**32 **  Return *CS*[1*..r*][1*..m*]

**Algorithm 2: **Greedy Placement Algorithm 1 (GPA1)

 **Input**: Contigs with associated scores *CS*[1*..r*][1*..m*]

 **Output**: Set of ordered contigs, *C*

 **begin**

**1 **  Create array of structure *struct*[1*..r*]

**2   for **(each contig, *c_i_*){

**3 **   Sort the matching score in increasing order

**4 **   Place *struct*[*i*]*.contig ← c_i_*

**5 **   Place *struct*[*i*]*.starting position ← starting_position*

**6 **   Place *struct*[*i*]*.ending position ← ending_position*

**7 **  }

**8 **  Sort the array of *struct*[1*..r*] with respect to *starting_position *in increasing order

**9   for **(each contig, *c_i _*in *struct*[1*..r*]){

**10    if **(*c_i _*is not overlapped with already placed contigs in *C*){

**11 **    Place the contig *c_i _*at the end of the list *C*

**12 **   }

**13 **  }

**14 **  Return *C*

#### Greedy placement algorithm 3 - GPA3

*GPA3 *takes as input the contigs and the ORM together with the output of Algorithm 1. If *m *is the number of ordered fragments in the ORM, then the number of scores (or mappings) associated with each contig will be *m*, as described in the previous section. The algorithm proceeds as follows: At first the matching scores associated with each contig are sorted individually in increasing order. The first position of the sorted list of each contig contains the minimum score (i.e., the best score) among all the scores.

We now sort the contigs based on their best scores. Let this sorted order be $C_{1}^{\prime \prime },C_{2}^{\prime \prime },\ldots ,C_{r}^{\prime \prime }$. Followed by this, we place the contigs in the ORM in this order. Specifically, we first try to place $C_{1}^{\prime \prime }$; Next we try to place $C_{2}^{\prime \prime }$; and so on. Note that for any given contig and a corresponding score, we know the starting fragment as well as ending fragment (in the ORM). While trying to place any contig *C*, we check if there will be any overlaps with any of the contigs already placed. If so, we move on to the next entry in *C*'s list and check if *C *can be placed based on the corresponding starting and ending fragments without any overlaps. We make a total of at most *d *such attempts to place *C *(where *d *is a user-defined parameter). If *C *cannot be placed successfully within these attempts, we drop *C *from further considerations and move on to the placement of the next contig.

A pseudocode of the algorithm can be found in Algorithm 4. Let *m *be the number of fragments in the optical map, and *r *be the number of contigs.

**Algorithm 3: **Greedy Placement Algorithm 2 (GPA2)

 **Input**: Contigs with associated scores *CS*[1*..r*][1*..m*], Depth, *d*

 **Output**: Set of ordered contigs, *C*

 **begin**

**1 **  Create array of structure *struct*[1*..r*]

**2   for **(each contig, *c_i_*){

**3 **   Sort the *matched_sites *in decreasing order

**4 **   Place the sorted list in *soretd_list *variable

**5 **   Place *struct*[*i*]*.contig ← c_i_*

**6 **   Place *struct*[*i*]*.matched_list ← matched_sites*

**7    for **(each matched sites, *mj *in the *matched_list*){

**8 **    Place *struct*[*i*]*.starting_position*[*j*] *← starting_position*[*j*]

**9 **    Place *struct*[*i*]*.ending_position*[*j*] *← ending_position*[*j*]

**10 **   }

**11 **  }

**12 **  Sort the array of *struct*[1*..r*] with respect to the greatest number of matched sites found in the first position of the *matched list*

**13   for **(each contig, *c_i _*in *struct*[1*..r*]){

**14    for **(*j ← *1; *j ≤ d*; *j ← j *+ 1){

**15     if **(*c_i _*is not overlapped with already placed contigs in *C*){

**16 **     Place the contig *c_i _*at the end of the list *C*

**17 **     Break

**18 **    }

**19 **   }

**20 **  }

**21 **  Sort the array *C *with respect to the starting position in increasing order

**22 **  Return *C*

The run time of lines 2-7 in Algorithm 4 is *O*(*mr*) as discussed above. Sorting contigs with respect to the least matching score takes *O*(*r*) time (line 8). Lines 13-20 take *O*(*rd*) time. In line 21 sorting contigs with respect to starting fragment takes *O*(*r*) time. Since *d *≪ *r *≪ *m*, the run time of Algorithm 4 is *O*(*mr*).

## Results and Discussions

To prove the effectiveness of our proposed algorithms we have done rigorous simulations on both real and synthetic datasets. The simulation results show that the algorithms are indeed scalable and efficient. We have also compared our algorithm with one of the best known algorithms [5]. Our algorithm outperforms the aforementioned algorithm in terms of run time, by more than two orders of magnitude, and accuracy. The run time of the scaffolding algorithm of [5] is *O*(*m*^2^*n*^2^*r*), where *m *is the number of fragments in the optical map, *r *is the number of contigs and *n *is the maximum number of fragments in any contig. In comparison, the run time of our algorithm is *O*(*mnr*). In this section we present our experimental results. All the programs have been run on an Intel Core i5 2.3 GHz machine with 4 GB of RAM.

**Algorithm 4: **Greedy Placement Algorithm 2 (GPA2)

 **Input**: Contigs with associated scores *CS*[1*..r*][1*..m*], Depth, *d*

 **Output**: Set of ordered contigs, *C*

 **begin**

**1 **  Create array of structure *struct*[1*..r*]

**2   for **(each contig, *c_i_*){

**3 **   Sort the matching score in increasing order

**4 **   Place the sorted list in *soretd_list *variable

**5 **   Place *struct*[*i*]*.contig ← c_i_*

**6 **   Place *struct*[*i*]*.score_list ← sorted_list*

**7    for **(each score, *sj *in the *sorted_list*){

**8 **    Place *struct*[*i*]*.starting_position*[*j*] *← starting_position*[*j*]

**9 **    Place *struct*[*i*]*.ending_position*[*j*] *← ending_position*[*j*]

**10 **   }

**11 **  }

**12 **  Sort the array of *struct*[1*..r*] with respect to the least score found in the first position of the *score_list*

**13   for **(each contig, *c_i _*in *struct*[1*..r*]){

**14    for **(*j ← *1; *j ≤ d*; *j ← j *+ 1){

**15     if **(*c_i _*is not overlapped with already placed contigs in *C*){

**16 **     Place the contig *c_i _*at the end of the list *C*

**17 **     Break

**18 **    }

**19 **   }

**20 **  }

**21 **  Sort the array *C *with respect to the starting position in increasing order

**22 **  Return *C*

### Real datasets

Real datasets are comprised of two strains of yersiniae bacteria, namely, *Yersinia pestis*, and *Yersinia enterocolitica*. The yersiniae are Gram-negative rods belonging to the family Enterobacteriaceae. They consist of 11 species of which three are pathogenic to humans. Those are *Yersinia pestis, Yersinia pseudo-tuberculosis*, and *Yersinia enterocolitica*. The genomic sequences of *Yersinia pestis *and *Yersinia enterocolitica *contain 4,653,728 bp and 4,615,899 bp, respectively. Each of the genomic sequences is randomly fragmented into a number of non-overlapping substrings/contigs of different lengths. We then permute the resulting contigs randomly to break the relative order existing among them. As we know the placement of the contigs when we generate them, we can easily detect whether our algorithms reconstruct the correct orderings from the randomly permuted contigs. To show the robustness of our proposed algorithms we introduce errors by discarding restriction sites with some probability. We also introduce errors by resizing, i.e., by increasing or decreasing the fragment sizes of the contigs.

We have generated 50, 100, 200, and 400 contigs from the genomic sequence of *Yersinia pestis *and 200, and 400 contigs from *Yersinia enterocolitica*. Accuracy is defined as the fraction of the contigs placed correctly. If a contig cannot be placed, i.e, if the placement overlaps with other contigs, we call it a conflict. On the contrary when the placement of a contig is out of order (i.e. when the contig is misplaced) we call it wrong placement. From Table 1 and Table 2 it is evident that if there are no errors in the datasets, the accuracy found by applying the different methods is in the range: [97%, 100%]. The less the number of contigs, the more accurate the resulting placement of the contigs are. In this case, the algorithms are more resilient with errors. It is also the case that GPA3 is more robust against the errors introduced in the datasets.

**Table 1:** Results for *Yersinia pestis*.

Contigs	Method	Missed probability	% Resize	Conflicts	Wrong placement	% Accuracy	Time (s)
50	GPA1	0.0	0	0	0	100.00	31.97
		0.1	5	0	0	100.00	29.45
		0.2	10	0	0	100.00	29.07
		0.3	20	27	1	44.00	25.99
	
	GPA2	0.0	0	0	0	100.00	34.10
		0.1	5	0	0	100.00	33.42
		0.2	10	0	0	100.00	30.83
		0.3	20	25	1	48.00	28.21
	
	GPA3	0.0	0	0	0	100.00	35.02
		0.1	5	0	0	100.00	32.41
		0.2	10	0	0	100.00	27.76
		0.3	20	12	2	72.00	27.24

100	GPA1	0.0	0	1	0	99.00	34.05
		0.1	5	4	0	96.00	31.23
		0.2	10	7	0	93.00	27.76
		0.3	20	45	6	49.00	25.92
	
	GPA2	0.0	0	1	0	99.00	31.44
		0.1	5	2	0	98.00	33.17
		0.2	10	4	2	94.00	26.18
		0.3	20	36	10	54.00	28.90
	
	GPA3	0.0	0	1	0	99.00	32.41
		0.1	5	0	0	100.00	30.10
		0.2	10	1	0	99.00	29.64
		0.3	20	27	6	67.00	29.04

200	GPA1	0.0	0	3	0	98.50	36.90
		0.1	5	8	0	96.00	33.28
		0.2	10	21	0	89.50	33.11
		0.3	20	69	4	63.5	29.61
	
	GPA2	0.0	0	3	0	98.50	33.56
		0.1	5	10	1	94.50	33.73
		0.2	10	19	3	89.50	34.29
		0.3	20	92	7	50.50	32.40
	
	GPA3	0.0	0	3	0	98.50	34.93
		0.1	5	5	0	97.50	35.96
		0.2	10	12	1	93.50	32.25
		0.3	20	52	5	71.5	32.16

400	GPA1	0.0	0	8	0	98.00	40.17
		0.1	5	20	2	94.50	35.00
		0.2	10	56	7	84.25	32.21
		0.3	20	120	15	66.25	30.47
	
	GPA2	0.0	0	8	0	98.00	34.77
		0.1	5	28	5	91.75	35.83
		0.2	10	47	25	82.00	33.15
		0.3	20	116	35	62.25	28.99
	
	GPA3	0.0	0	7	0	98.25	37.64
		0.1	5	18	0	95.50	31.70
		0.1	5	29	8	90.75	31.50
		0.3	20	162	21	76.75	31.70

**Table 2:** Results for *Yersinia enterocolitica*.

Contigs	Method	Missed probability	% Resize	Conflicts	Wrong placement	% Accuracy	Time (s)
200	GPA1	0.0	0	0	0	100.00	43.37
		0.1	5	5	0	97.50	43.97
		0.2	10	18	0	91.00	38.92
		0.3	20	92	4	51.00	28.32
	
	GPA2	0.0	0	0	0	100.00	46.41
		0.1	5	3	0	98.50	45.47
		0.2	10	11	6	91.50	32.71
		0.3	20	84	10	53.00	32.29
	
	GPA3	0.0	0	0	0	100.00	41.10
		0.1	5	6	2	96.00	43.61
		0.2	10	11	0	94.50	40.41
		0.3	20	57	7	68.00	31.87

400	GPA1	0.0	0	9	0	97.75	46.67
		0.1	5	17	1	95.50	45.02
		0.2	10	45	1	88.50	37.00
		0.3	20	111	18	67.75	32.95
	
	GPA2	0.0	0	10	1	97.25	46.66
		0.1	5	26	4	92.50	49.04
		0.2	10	50	22	82.00	33.21
		0.3	20	135	26	59.75	31.90
	
	GPA3	0.0	0	9	0	97.75	43.89
		0.1	5	15	0	96.25	36.04
		0.2	10	29	5	91.50	33.53
		0.3	20	54	23	80.75	33.04

**Figure 1:**
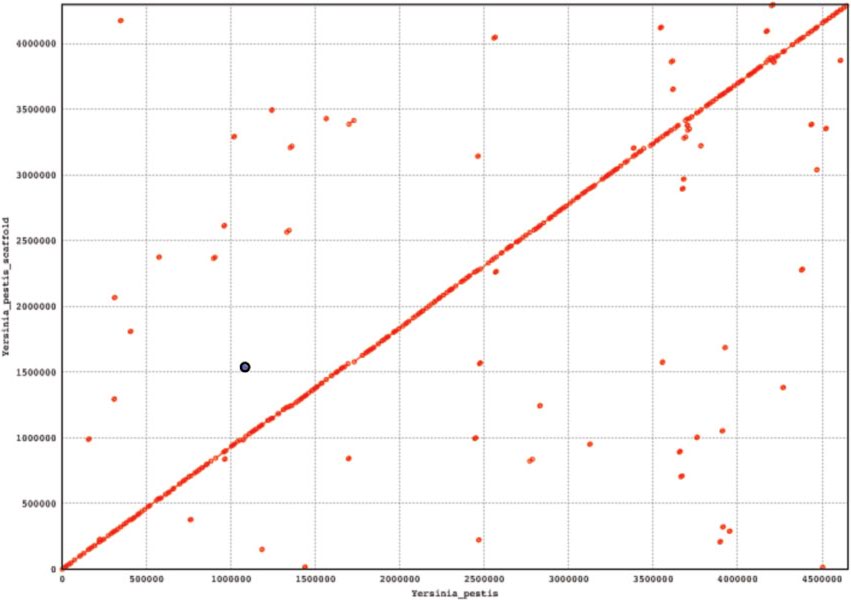
**Aligning ordered contigs onto the *Yersinia pestis***.

**Figure 2:**
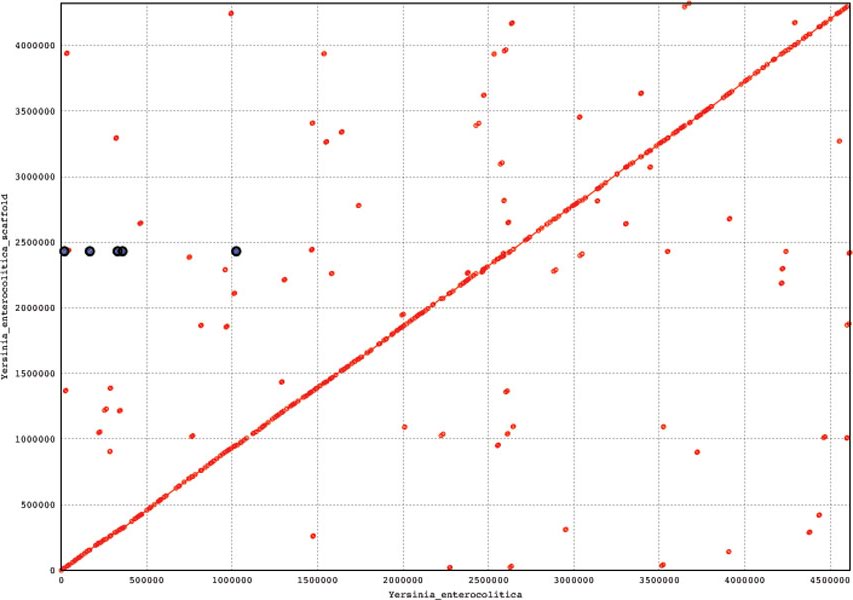
**Aligning ordered contigs onto the *Yersinia enterocolitica***.

To simulate practical scenarios, we have randomly generated reads of size 100 bp each from the two Yersinia strains. Contigs were created employing the String Graph Assembler (SGA) [15]. These contigs were then ordered using GPA2. After ordering we concatenated the ordered contigs to find the scaffold. As the sequences are very long, it is infeasible to calculate the edit distance between the original sequence and resulting scaffold. So, the genomic sequence and the corresponding scaffold are aligned using MUMmer [16]. The acronym "MUMmer" comes from "Maximal Unique Matches", or MUMs. It is based on the suffix tree data structure designed to find maximal exact matches of two input sequences. In Figure 1 we have aligned ordered contigs of *Yersinia pestis *onto the original sequence of *Yersinia pestis*. We have aligned ordered contigs of *Yersinia enterocolitica *onto the original sequence of *Yersinia enterocolitica*. The plots [Please see Figure 1 and 2] represent the set of all MUMs between the two input sequences. Forward MUMs are plotted as red lines/dots while reverse MUMs are plotted as blue lines/dots (encircled). A line of dots with unit slope represents an undisturbed segment of conservation between the two sequences, while a line of dots with negative unit slope represents an inverted segment of conservation between the two sequences. As is evident, the alignments ordered contigs (i.e. scaffold) are nicely placed onto the original sequences. The coverage of these two alignments is approximately 92% which proves the effectiveness of our algorithms.

### Synthetic datasets

We have generated four genomic sequences of various sizes by choosing each character randomly from a uniform distribution. We generated reads of size 100 bp from each of the datasets such that the average coverage of the reads to a particular position of the sequence is around 5. Reads were generated by taking substrings of size 100 bp from randomly selected positions in the sequence. SGA [15] was used to generate contigs from the reads. The contigs were then ordered using our algorithms. ORM is created *in silico *by choosing a 4-bp long sequence acting as a restriction enzyme. The ordered fragment sizes of each contig are also created by employing the same procedure stated above. After getting the scaffold we calculate the edit distance between the original sequence and the resulting scaffold. It is intuitive that if the placement of the contigs in the scaffold is correct, then the following statement holds: *|Size*(*original_sequence*) *− Size*(*constructed_sequence*)*| ≈ edit_distance*(*original_sequence, constructed_sequence*). Our simulation results show that this is indeed the case [Please see Table 3].

**Table 3:** Results for simulated data.

Length	Contigs	Method	Placed	Observed length	Difference	Edit dist	Coverage	Time (s)
1 *× *10^5 ^*bp*	7	GPA1	6	84689	15311	15483	84.69%	0.40
		
		GPA2	6	84689	15311	15483	84.69%	0.45
		
		GPA3	6	84689	15311	15483	84.69%	0.37

3 *× *10^5 ^*bp*	34	GPA1	26	259619	40381	40923	86.54%	1.95
		
		GPA2	26	281905	18095	18917	93.97%	2.07
		
		GPA3	32	260662	39338	86220	86.89%	2.10

5 *× *10^5 ^*bp*	52	GPA1	39	445727	54273	55210	89.15%	4.67
		
		GPA2	39	454376	45624	50185	90.88%	5.46
		
		GPA3	38	431582	68418	69285	86.32%	4.67

7 *× *10^5 ^*bp*	53	GPA1	43	571908	128092	129160	81.70%	8.62
		
		GPA2	45	656139	45624	48593	93.73%	8.27
		
		GPA3	50	586588	113412	143189	83.80%	8.17

### Comparison

We have compared our algorithms with one of the the best known algorithms existing in the literature [5]. The simulation results show that our proposed algorithms are superior in terms of both run time as well as accuracy. As the size of the sequence is increased more and more, our algorithms are faster and faster than [5]. We have compared our proposed algorithms with [5] by using synthetic datasets. The ground truth of exact ordering of contigs is unknown in the case of real datasets as we do not know the placement of the contigs in prior. As optimal ordering is NP-hard, computationally it is impossible to find the correct placement when the number of contigs is large. So, to compare with [5] we have generated 4 artificial sequences of various sizes. 50 contigs were generated from each of the sequences. Contigs generation process is described in Section. Accuracy is calculated as the fraction of contigs placed correctly. As is evident from the simulation results, our algorithms are two orders of magnitude faster and our placements are also better [Please see Table 4] than [5]. In some cases we did not calculate the accuracy as it was taking an indefinite amount of time compared to our algorithms. '-' indicates this issue in Table 4.

**Table 4:** Comparisons.

Length	Method	Correctly placed	Accuracy	Time (s)
5 *× *10^5 ^*bp*	GPA1	49	98.00%	5.87
	
	GPA2	49	98.00%	4.65
	
	GPA3	49	98.00%	4.62
	
	Nagarajan et al. [5]	30	60.00%	1620

6 *× *10^5 ^*bp*	GPA1	50	100.00%	8.52
	
	GPA2	50	100.00%	7.12
	
	GPA3	50	100.00%	7.12
	
	Nagarajan et al. [5]	32	64.00%	14400

7 *× *10^5 ^*bp*	GPA1	49	98.00%	8.79
	
	GPA2	49	98.00%	8.19
	
	GPA3	49	98.00%	8.48
	
	Nagarajan et al. [5]	-	-	-

8 *× *10^5 ^*bp*	GPA1	50	100.00%	11.77
	
	GPA2	50	100.00%	11.70
	
	GPA3	50	100.00%	10.64
	
	Nagarajan et al. [5]	-	-	-

## Conclusions

Contig assembly is a very challenging task. In *de novo *assembly it is one of the most important steps to construct an entire genomic sequence from millions of reads produced by the sequencers. A series of algorithms has been proposed in this paper to order the contigs. ORM is used to calculate matching scores between the sequence and contigs. Contigs are then placed so that the overall cumulative matching scores are minimized. We have performed rigorous simulations on both real and synthetic datasets. The results show that our algorithms are efficient in terms of both run time and accuracy.

## Competing interests

None

## Authors' contributions

SS and SR have come up with the algorithms. SS has implemented the algorithms. The results have been analyzed and the algorithms have been optimized by SS and SR. SS and SR have written the paper.

## Declarations

The publication charges for this article were funded by the NIH grant R01-LM010101.

This article has been published as part of *BMC Genomics *Volume 15 Supplement 5, 2014: Selected articles from the Third IEEE International Conference on Computational Advances in Bio and Medical Sciences (ICCABS 2013): Genomics. The full contents of the supplement are available online at http://www.biomedcentral.com/bmcgenomics/supplements/15/S5.
